# In Vitro Examinations of Cell Death Induction and the Immune Phenotype of Cancer Cells Following Radiative-Based Hyperthermia with 915 MHz in Combination with Radiotherapy

**DOI:** 10.3390/cells10061436

**Published:** 2021-06-08

**Authors:** Michael Hader, Simon Streit, Andreas Rosin, Thorsten Gerdes, Martin Wadepohl, Sander Bekeschus, Rainer Fietkau, Benjamin Frey, Eberhard Schlücker, Stephan Gekle, Udo S. Gaipl

**Affiliations:** 1 Department of Radiation Oncology, Universitätsklinikum Erlangen, Friedrich-Alexander-Universität Erlangen-Nürnberg (FAU), 91054 Erlangen, Germany; michael.hader@uni-bayreuth.de (M.H.); rainer.fietkau@uk-erlangen.de (R.F.); benjamin.frey@uk-erlangen.de (B.F.); 2 Translational Radiobiology, Department of Radiation Oncology, Universitätsklinikum Erlangen, Friedrich-Alexander-Universität Erlangen-Nürnberg (FAU), 91054 Erlangen, Germany; 3 Chair for Ceramic Materials Engineering, Keylab Glastechnology, University of Bayreuth, 95447 Bayreuth, Germany; andreas.rosin@uni-bayreuth.de (A.R.); thorsten.gerdes@uni-bayreuth.de (T.G.); 4 Institute of Process Machinery and Systems Engineering (iPAT), Friedrich-Alexander-Universität Erlangen-Nürnberg (FAU), 91058 Erlangen, Germany; sl@ipat.uni-erlangen.de; 5 Biofluid Simulations and Modeling, Fachbereich Physik, University of Bayreuth, 95447 Bayreuth, Germany; simon-streit@t-online.de (S.S.); stephan.gekle@uni-bayreuth.de (S.G.); 6 Dr. Sennewald Medizintechnik GmbH, 81829 Munich, Germany; martin.wadepohl@sennewald.de; 7 ZIK Plasmatis, Leibniz Institute for Plasma Science and Technology, Felix-Hausdorff-Str. 2, 17489 Greifswald, Germany; sander.bekeschus@inp-greifswald.de

**Keywords:** hyperthermia, radiofrequency, heat source, immune modulatory effects, multimodal tumor treatment, immune checkpoint inhibitors

## Abstract

Multimodal tumor treatment settings consisting of radiotherapy and immunomodulating agents such as immune checkpoint inhibitors are more and more commonly applied in clinics. In this context, the immune phenotype of tumor cells has a major influence on the anti-tumor immune response as well as the composition of the tumor microenvironment. A promising approach to further boost anti-tumor immune responses is to add hyperthermia (HT), i.e., heating the tumor tissue between 39 °C to 45 °C for 60 min. One key technique is the use of radiative hyperthermia systems. However, knowledge is limited as to how the frequency of the used radiative systems affects the immune phenotype of the treated tumor cells. By using our self-designed in vitro hyperthermia system, we compared cell death induction and expression of immune checkpoint molecules (ICM) on the tumor cell surface of murine B16 melanoma and human MDA-MB-231 and MCF-7 breast cancer cells following HT treatment with clinically relevant microwaves at 915 MHz or 2.45 GHz alone, radiotherapy (RT; 2 × 5 Gy or 5 × 2 Gy) alone or in combination (RHT). At 44 °C, HT alone was the dominant cell death inductor with inactivation rates of around 70% for B16, 45% for MDA-MB-231 and 35% for MCF-7 at 915 MHz and 80%, 60% and 50% at 2.45 GHz, respectively. Additional RT resulted in 5–15% higher levels of dead cells. The expression of ICM on tumor cells showed time-, treatment-, cell line- and frequency-dependent effects and was highest for RHT. Computer simulations of an exemplary spherical cell revealed frequency-dependent local energy absorption. The frequency of hyperthermia systems is a newly identified parameter that could also affect the immune phenotype of tumor cells and consequently the immunogenicity of tumors.

## 1. Introduction

Cancer is the first or second leading cause of premature death in over 100 countries around the world [1]. Interestingly, there is a correlation between the Human Development Index (HDI) and the cancer incidence rate in a country, mainly due to the behavior and lifestyle in these countries. When the HDI increases worldwide, the number of cancer cases also increases. As a result, much research needs to be done to find new and better treatment options in this area, with a focus on a good cost-benefit ratio. In this context, a study by PwC predicts that per capita costs will need to decrease by approximately 6.5–27.5% between 2018 and 2030 in order to provide treatment for all populations [2].

Currently, a combination of the following treatment options is commonly used: surgery, radiotherapy (RT), chemotherapy, and increasingly immunotherapy such as immune checkpoint inhibitors (ICI). Because of their high level of commitment and good results, these methods are often referred to as the four pillars of cancer treatment. With ICI, however, the response rate is mostly dependent on the tumor, its microenvironment, and the treatment scheme. In order to improve the response rate and ultimately the clinical outcome, other methods have come into focus in recent years [3,4]. A fairly old, but promising, method with successful results in phase I-III studies is radiative-based hyperthermia. Hyperthermia should always be seen as an additive method used in combination with the options already available. The core idea of hyperthermia is the targeted heating of cancer tissue to temperatures between 39 °C and 45 °C. In various clinical studies, a significantly higher tumor regression has been reported using RT or radio(chemo)therapy in combination with hyperthermia [5,6].

To apply clinical hyperthermia, different technologies can be considered depending on the size and location of the tumor. Often a subdivision in whole-body, regional or local hyperthermia is made first; then further differentiation is made regarding the depth at which it is operated upon. For regions up to a depth of 4 cm under the skin, it is classified as superficial, otherwise as deep hyperthermia. Depending on this differentiation, specific heating means are used to achieve the temperature rise. Conventional heating (CH) takes place by conducting heat through a circulating liquid, which can be applied externally or internally (e.g., intraperitoneal hyperthermic chemoperfusion, HIPEC) [7,8,9,10]. Another mean is to use electromagnetic waves in the range of radio- or microwaves (13.45 MHz to 2.45 GHz) to heat the tissue. Microwave heating (MH) offers several advantages over conventional heating due to the higher localization of the heating and the ability to be applied non-invasively. Even deep-seated tissue and various shapes can be precisely heated with multi-antenna systems and thoroughly controlled interference patterns of the electromagnetic waves [11,12]. A previous study showed that the inactivation rate of B16 melanoma cells in vitro is considerably higher under MH than CH at the same treatment temperature [13]. At 44 °C, the inactivation under CH is reported to reach 12%, while the inactivation led under MH to an increase of inactivation rates to over 70%. This implies that electromagnetic effects could also play a role in inactivating the cells.

The formation of pores due to an electric field is an example of such an electromagnetic effect [14], but the structure and function of other proteins could also be influenced by, e.g., immune checkpoint molecules on the cells’ surface or the release of danger signal molecules such as heat shock protein 70 (HSP70). Our work aims to provide answers to the effects of microwave radiation at cellular level using techniques from immunobiology, process engineering and molecular dynamic simulations. This could help to find new therapeutic opportunities, both for hyperthermia heating systems and for multimodal tumor treatments. In this work we present for the first time an ex vivo microwave heating system operating with 915 MHz which is clinically used for superficial heating of, e.g., breast wall recurrences or melanoma, and how this treatment affects the immune phenotype of tumor cells.

## 2. Materials and Methods

### 2.1. Closed-Loop System

**Figure 1:**
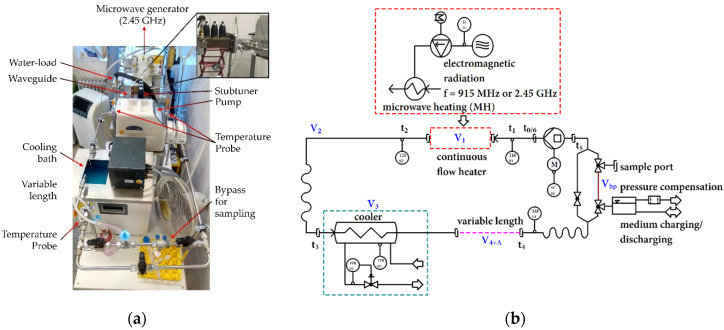
Closed-loop system: (**a**) real system structure with exemplary 2.45 GHz heating unit; (**b**) RI-flowchart of the modular heating system with sample bypass, flow heater and cooler. Conceptually based and adapted from [14].

Ex vivo heat treatment of the cell suspension was performed in a self-designed stainless-steel lab-scale closed-loop system under sterile conditions (Figure 1a,b). The closed-loop system was designed with modular heating units, i.e., a microwave heating unit at 915 MHz or 2.45 GHz, to achieve target temperatures (*T*_target_) in the range from 37 °C to 44 °C. To mimic blood perfusion, the flow rate was kept constant at 2 mL/s. Within the closed-loop system, temperature was monitored at the inlet (TIR01) and outlet (TIR02) of the respective heating unit as well as within the cooler (TIR03) and at the end of the variable length (TIR04). Furthermore, the level, i.e., power, of the microwave units was controlled and measured.

**Figure 2:**
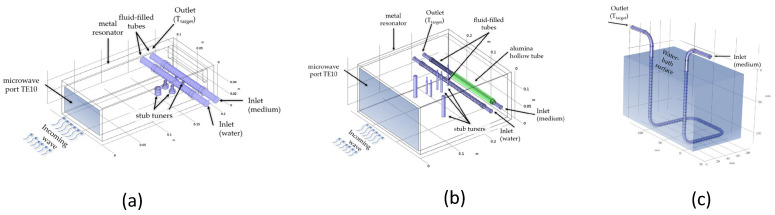
Microwave heating units within the closed-loop system: (**a**) 2.45 GHz microwave heating unit and (**b**) self-designed 915 MHz microwave heating for microwave heating (MH). Both microwave heating units have TE10 ports, two horizontally aligned quartz-glass tubes, one for the cell suspension (rear tube), and one as variable dummy absorber (front tube). In addition, stub tuners are used for optimizing and controlling the electromagnetic energy absorption of the cell suspension; (**c**) cooling is achieved by a stainless-steel loop immersed into a controllable water-bath.

The 2.45 GHz microwave setup consisted of a 2 kW-microwave generator (TM A20 S1) with a circulator for catching and dissipating the reflected power. For the 2.45 GHz experiments, a custom-made rectangular waveguide (MW1002E-260EC, WR-430/R26, 86 mm × 43 mm) was used with two horizontally aligned quartz-glass tubes (*d*_i_ = 10.2 mm, *d*_a_ = 13.0 mm, tube length 86 mm for media and water load) at the applicator’s closed end, and with manual stub tuners for fine-tuning the energy absorption (Figure 2a). For the 915 MHz experiments, a standard waveguide (WR-975, 247 mm × 124 mm) was modified with two horizontally aligned quartz-glass tubes (*d*_i_ = 10.2 mm, *d*_a_ = 13.0 mm, tube length 247 mm for both the media and water load) (Figure 2b). To achieve the same irradiated area/volume as for the 2.45 GHz system, an alumina hollow tube with a length of 161 mm covered the media load partially (Figure 2b**,** green). The respective media volume of the heating unit, *V*_1_, was kept constant at 6.88 cm^3^. *V*_2_ was set to 25 cm^3^ and *V*_3_ was 8.50 cm^3^. The total system volume, *V_m_*, was set to 129 cm^3^_,_ and *V*_4+Δ_ was 88.62 cm^3^. The effective total volume, *V*_m,eff_, is in fact 119 cm^3^ as 10 cm^3^ (*V_bp_*) are reserved for bypass and sampling. In case of 915 MHz MH a volume reduction of 13.80 cm^3^ in the variable length was necessary for the 915 MHz setup as the total media volume in the 915 MHz heating unit was 20.68 cm^3^ in comparison to 6.88 cm^3^ in the other system operating at 2.45 GHz. For the self-designed stainless-steel cooling-loop (d_i_ = 4 mm, d_a_ = 6 mm), a total volume of 7.49 cm^3^ was used to achieve a cooling capacity for all treatment conditions (Figure 2c). During treatment, the media circulates in the loop while being recurringly heated and cooled. The effective treatment time, *t*_eff_, can be expressed by the number of cycles, *N*_0_, and the net heating time per cycle, ${\overset{-}{\underset{\;}{t}}}_{heat}$. Latter can also be expressed by the time of circulation, *t*_cycle_, and the ratio of the sum of *V*_1_ and *V*_2_, and the total medium volume, *V*_m,eff_. The time of circulation, *t*_cycle_, was 59.5 s at a flow rate of 2.0 mL/s ($V_{m,\mathrm{eff}}/{\dot{V}}_{m}$). The number of cycles, N_0_, was 242 turns for a total time of *t*_eff_ = 60 min. The effective heating time is about ¼ (1:3.73) of the total treatment time, i.e., 60 min of effective heating is applied within 4 h (14,400 s).

### 2.2. Numerical Simulations of the Heating Units

As the temperature of the cell suspension can only be measured at the inlet and outlet (Figure 1 and Figure 2), the real temperature distribution within the two microwave heating units is unknown. Therefore, numerical simulations of the electromagnetic field distribution and the coupled fluid-flow and temperature profiles within the heating area were performed with the commercial software COMSOL^®®^ Multiphysics^©^ 5.5. In all simulations, the medium flow rate, ${\dot{V}}_{m}$, was kept constant at 2 mL/s and the target temperature, *T*_target_, was varied between 37 °C and 44 °C. In electromagnetic heating processes, the most relevant material parameter is the complex permittivity, consisting of the real part, $\varepsilon_{r}^{\prime }$, and the imaginary part, $\varepsilon_{r}^{\prime \prime }$. The permittivity is a function both of temperature and frequency (Table 1). As a simplification we took published permittivity data of pure water for our simulations because the dielectric parameters of the dilute aqueous cell media were not available. We could validate this assumption experimentally by calibration tests. More details about our simulation and measurement concept are described elsewhere [14].

### 2.3. Cultivation and Inactivation of Tumor Cells

All cancer cell lines were cultivated at 37 °C in 5% CO_2_ and 90% humidity under sterile conditions. The two human breast cancer cell lines, MDA-MB-231 and MCF-7, were grown in Dulbecco’s modified Eagle’s medium and the murine B16 melanoma cell line in RPMI 1640, each supplemented with 10% fetal bovine serum, 1% sodium pyruvate, 2 mM glutamine, 100 U/mL of penicillin and 100 µg/mL of streptomycin. MDA-MB-231 cell line is triple negative, i.e., ER negative, PgR negative, p53 mutated and caspase-3 intact, while MCF-7 cells are ER positive, PgR positive, p53wt and deficient of caspase-3 [17,18,19,20]. Both, splitting and harvesting the cells was conducted by trypsination (10% trypsin in DPBS) on a heating plate for 2–3 min.

**Figure 3:**
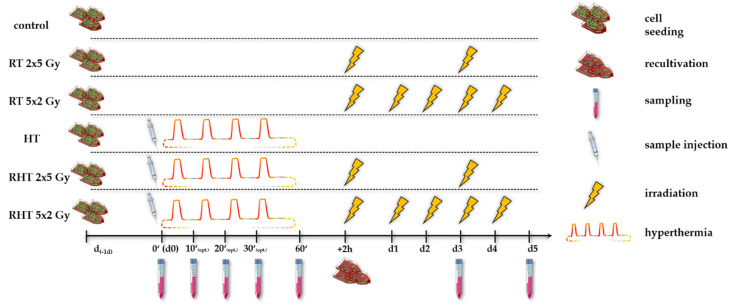
Experimental treatment set-up: The cells were seeded cell line-dependent one day prior to the treatment (*d*_-1d_) for not more than 90% of confluency during the whole treatment. Standard sampling in all arms was performed on day 0 (*d*0), *d*3 (72 h) and *d*5 (120 h). In the HT and combinatory arm, i.e., radiotherapy & hyperthermia (RHT), sample injection of 1 × 10^7^ cells into the HT system was performed at time point *d*0 (0′), followed by an effective heating session of at most 60 min (60′). After the HT treatment, cells were recultivated into 75 cm^2^ t-flasks according to their subsequent treatment. Irradiation in the radiotherapy arm (RT) and combinatory RHT arm was performed in 75 cm^2^ t-flasks with clinically relevant doses of either 2 × 5 Gy (hypo) or 5 × 2 Gy (normo) at time point +2 h. Irradiation in the respective RT and RHT arms was always performed at the same time [14].

Inactivation of the tumor cells was performed according to Figure 3. One day prior to the respective treatment (d_-1d_), the cells were seeded according to the respective cell line, so that the confluency never exceeded 90%. At time point *d*0 (0′), both for HT and the combinatory arm, i.e., HT + RT (RHT), sample injection of 1 × 10^7^ cells was followed by an effective heating time of at most 60 min (60′). During the (R)HT session at a constant flow rate of 2 mL/s at *T*_target_ = 39 °C, 41 °C or 44 °C, optional sampling at time points 10 min (10′), 20 min (20′) and 30 min (30′) could be done. In this paper, we only focus on 30′ as optional time point. The total treatment time is four times more than the effective treatment time, i.e., 60 min of effective heating means 4 h of cyclic heating in total. The cells were split into 75 cm^2^ t-flasks according to their subsequent treatment. For the RHT arms, irradiation was performed for 2 h after HT treatment, as common in multimodal treatments [21].

### 2.4. Flow Cytometry Analysis for the Detection of Cell Death Forms and Immune Checkpoint Molecule Expression

Multicolor flow cytometry (Beckman EPICS^®®^ XL^™^ and Cytoflex™ S) was used to detect cell death forms by AnnexinV-FITC (AxV)/propidium-iodide (PI) staining and to determine immune checkpoint molecules (ICM) by antibody staining. 

For cell death detection, PI penetrates only into cells that have lost their membrane integrity. Due to the binding mechanism of PI with DNA, the intensity of the PI signals allows a first differentiation between primary necrosis and secondary necrosis, since primary necrotic cells have the full DNA content as viable cells and secondary necrotic cells derive from apoptotic ones that already have degraded and shedded DNA via apoptotic bodies [22]. By co-staining with FITC-conjugated AnnexinV as described in [23], apoptotic cells that undergo controlled cell death and express phosphatidylserine (PS) on the outer membrane leaflet, can be differentiated from necrotic cells as their membrane integrity is still intact. To sum up, AxV^+^/PI^-^ cells are apoptotic, AxV^+^/PI^++^ cells are primary and AxV^+^/PI^+^ are secondary necrotic. AnnexinV/PI-staining was always performed in duplicates according to in-house standard operating procedures (SOP). For the EPICS XL system, 400 µL Ringer^®®^ solution stained with 1.0 µg/mL of PI and 0.5 µg/mL of FITC-labeled AnnexinV was resuspended with 100,000 cells/tube. For the Cytoflex S system, 100,000 cells/well were resuspended in 100 µL Ringer^®®^ solution stained with 4.0 µg/mL of PI and 2.0 µg/mL of FITC-labeled AnnexinV.

For analysis of immune checkpoint molecule expression, 100,000 cells/well were incubated with 100 µL of the cell line specific antibody staining solution (Table 2) for 30 min in the dark at 4 °C. In order to avoid an overlap of fluorescence peaks, two Mastermixes #1 and #2 were designed using the open source FluoroFinder^®®^ software. All antibody concentrations were tested according to manufacturer’s instruction and finally titrated for the human and murine cell lines. To distinguish viable from dead cells, Zombie NIR (human, murine#1) or Zombie Yellow (murine#2) at a concentration of 0.1 µL/well was used. The mean fluorescence intensity of stained samples was subtracted from unstained mock-treated samples that contained only FACS-buffer and Zombie NIR/Yellow.

### 2.5. Single Cell and Molecular Dynamics Simulation of DNA-Components

**Figure 4:**
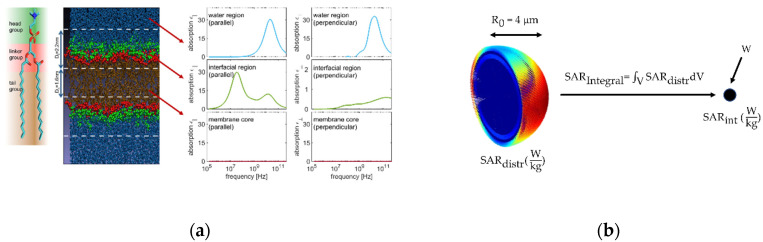
(**a**) The phospholipid DPPC forms a bilayer in a simulation box with water. Spatial dependent absorption spectra are calculated from the equilibrium simulations [25]; (**b**) 3D-SAR distribution for an exemplary small cell is integrated and reduced to a single point absorber.

An electric field that penetrates a single cell affects the electric potential inside the cell and the absorbed heat. Both can be described mathematically by Maxwell‘s equations and dielectric boundary conditions. To simplify the highly complex structure of a single cell, only soluble, bulk water and three regions of a dipalmitoyl-phosphatidylcholine (DPPC) phospholipid were modeled including a polar head, linker and tail group (Figure 4a). The volume specific absorbed heat, *W*, which is used in hyperthermia treatment and better known as the specific absorption rate, SAR in W/kg, can be calculated using Equation (1):

(1) $$W=SAR=-\pi \frac{f}{\rho }Im\left({\vec{E}}^{0*}\cdot {\vec{D}}^{0}\right)$$

with the frequency *f* of the applied electromagnetic wave, the density *ρ* of the corresponding medium, the complex conjugated amplitude $\vec{E}$^0*^ of the electric field and $\vec{D}$^0^ as the amplitude of the dielectric displacement field. To solve Equation (1), both, the electric field and dielectric displacement field need to be calculated first. Since both are connected via the permittivity, it is sufficient to work out only one field. As with similar problems, one can use the electric potential as a starting point. The electric potential must satisfy the tensorial Laplace equation

(2) ∇ · ϵ∇ϕ=0.

By applying a homogenous electric field ${\vec{E}}_{ext}={\vec{E}}_{ext}^{0}e^{i\omega t}$, with ${\vec{E}}_{ext}^{0}$ and frequency ω, at the outer shell, the resulting electric potential in the *i*-th shell is described according to [24] as

(3) $$\phi_{i}\left(r,\;\theta ,t\right)=A_{i}e^{i\omega t}r^{\delta_{i}}cos\theta +B_{i}e^{i\omega t}\frac{1}{r^{\delta_{i}+1}}cos\theta$$

with the complex constants A_i_, B_i_ and $2\delta_{i}=-1+\left(1+8\frac{\epsilon_{∥,i}}{\epsilon_{⊥,i}}\right).$ While *δ*_i_ can be directly derived from absorption spectra (Figure 4a), A_i_ and B_i_ need to be calculated for each region. After applying appropriate boundary conditions, e.g., the outermost shell potential is given by the external field $\phi (r,\theta ,t)={\vec{E}}_{ext}^{0}e^{i\omega t}R_{N}cos\theta$, a linear equation system can be solved with help of LU-decomposition algorithm in MATLAB. A more detailed description of this calculation method is published in [25]. Finally, for a better comparison between different parameters (cell radius, frequency), an integration of the SAR-distribution over the whole cell was performed according to Equation (4), which is conceptionally illustrated in Figure 4b.

(4) $${\mathrm{SAR}}_{\mathrm{Integral}}=\int_{0}^{\phi =2\pi }\int_{0}^{\theta =R}\int_{0}^{{r=R}_{0}}\mathrm{SAR}\left(r,\theta \right)r^{2}\;\sin\theta \;\mathrm{dr}\;d\theta \;d\phi$$

## 3. Results

The use of incubators or warm-water baths for ex vivo hyperthermia (HT) is easy to perform but does not focus on the clinically relevant radiative-based heating technique. However, radiative-based in vitro hyperthermia has some limitations, such as the use of t-flasks in a microwave chamber. Here, local hot-spots are often observed due to inhomogeneities in the distribution of the electromagnetic field, and sufficiently accurate online temperature monitoring is hardly possible. Further, frequencies of microwaves that are used in clinical devices should be used for the preclinical examinations. We therefore focused in this work on microwave radiation-based HT at 915 MHz and used our self-designed closed-loop media flow system with modular heating devices (Figure 1). After the development of a 915 MHz heating unit and adaption in the modular system, we compared cell death forms after 915 MHz with 2.45 GHz hyperthermia. The latter frequency was used in preceding work focusing generally on differences between warm-water and microwave heating [14]. Special focus was set at early time points 0 min (0′), 30 min (30′), and 60 min (60′)) after HT application and later on, as well as on day 3 (*d*3) and day5 (*d*5), for analyses of the effects of normo- and hypo-fractionated radiotherapy alone and in combination with 915 MHz hyperthermia (RHT).

### 3.1. Self-Developed 915 MHz Hyperthermia Heating System Allows Reproducible Treatment without Excessive Hot-Spots

**Figure 5:**
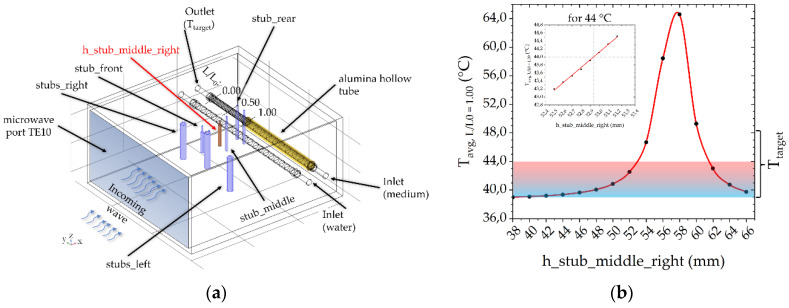
(**a**) 915 MHz cavity with alumina hollow tube (yellow) and stub tuners (blue) with TE10-port. (**b**) Relationship between varied height of h_stub_middle_left (red) and average temperature at outlet, T_avg,L/L0 = 1.00_, with the rest of the stub tuners fixed. The marked area describes the temperature range within the experiments between 39 °C and 44 °C.

To perform hyperthermia at 915 MHz in our modular closed-loop system, we modified a TE10-port waveguide (WR-975, 247 mm × 124 mm) into a resonator cavity (Figure 5a). For this, since the propagation of the electromagnetic field and the temperature distribution in the two fluid tubes is difficult to determine without numerical calculation tools, we used COMSOL Multiphysics. First, we inserted two horizontally aligned quartz-glass tubes (*d*_i_ = 10.2 mm, *d*_a_ = 13.0 mm, tube length 247 mm for both the media and water load). For physical reasons, the 915 MHz waveguide is wider by a factor of 2.67 than the waveguide for 2.45 GHz, so a shielding alumina hollow tube (marked in yellow in Figure 5a) was placed over part of the media line. Thus, the effective heating volume, *V_1_*, is identical with the 2.45 GHz applicator, i.e., 6.88 cm^3^. Then, different tuning pins (marked in blue in Figure 5a) were positioned in the x–y field and additionally their height (z-direction) was varied. Tuning pins were necessary to achieve enough energy coupling into medium and water. Without any tuning pins, the temperature difference in the media tube would be only 0.5 °C at 500 W. Additionally, an optimization function was implemented to minimize temperatures above *T_target_* in the axial and longitudinal section (more in Section 3.2). Finally, we focused on a simple controllability of T_target_, where we defined the height of the middle_right_stub tuner as single variable. As shown in Figure 5b, the required temperature range between 39 °C and 44 °C can be controlled well by increasing the height of the middle right stub tuner.

**Figure 6:**
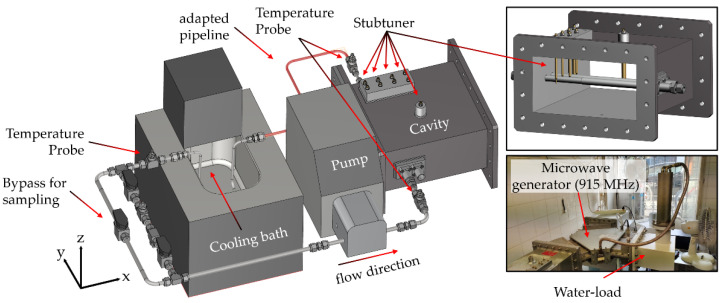
Closed-loop system with a 915 MHz heating unit (cavity) to heat up the cells between 39 °C and 44 °C.

Since the volume in the 915 MHz heating unit was 13.80 cm^3^ larger than in the 2.45 GHz heating unit, and the treatment should remain as similar as possible, modifications were done. On the one hand, an adapted pipeline was installed after the outlet from the heating unit to gain space (red area), and on the other hand, the bypass after the cooling bath was removed. As a result of the modification, the effective total volume, *V_m,eff_*, as well as the effective heating time, *t_eff_*, per cycle remained identical to the 2.45 GHz system. Figure 6 shows the complete system with pump, cooling bath and 915 MHz heating unit as well as the microwave generator and the water load.

### 3.2. Numerical Simulations to Demonstrate Comparable Heating Conditions in Both Microwave Cavities

**Figure 7:**
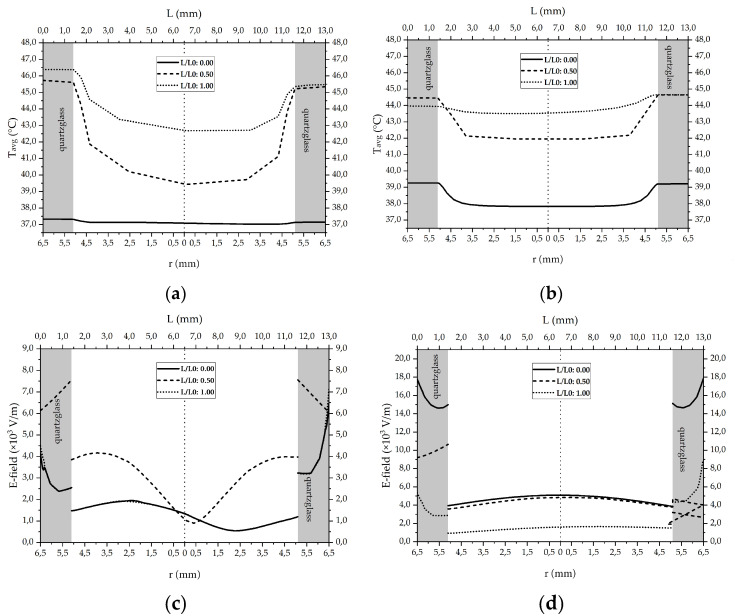
Simulated radial temperature profiles for (**a**) 2.45 GHz and (**b**) 915 MHz and simulated electric field distribution for (**c**) 2.45 GHz and (**d**) 915 MHz at the beginning, middle and end of the exposed tube piece. Process conditions in this example were *T*_target_ = 44 °C and ${\dot{V}}_{m}$ = 2 mL/s at *P*_MW_ = 100 W at 2.45 GHz, or *P*_MW_ = 500 W at 915 MHz, respectively. Additional information about the heating systems used for simulation and experiments is provided in Section 2.1 and Section 2.2.

Clinical treatment planning for hyperthermia includes the distribution of the electromagnetic field (V/m), the specific absorption rate (W/kg or W/m^3^), the temperature distribution (°C) and finally biological correlations [26]. For both in vitro and in vivo studies, data are sometimes missing or insufficiently collected. In order to obtain detailed information about the physical conditions inside the cell media carrying quartz-glass tubes at 915 MHz and 2.45 GHz, numerical simulations were carried out (Figure 2a,b). Figure 7a,b illustrate the calculated radial temperature curves within the tubes at 2.45 GHz (Figure 7a), and at 915 MHz (Figure 7b) for three horizontal intersecting lines, which are normalized by total length, *L*(*z*)/*L*_0_. The heat treatment and fluid flow conditions were determined by *T*_target_ = 44 °C and ${\dot{V}}_{m}$ = 2 mL/s.

Figure 7 shows the horizontal temperature and electric field profiles inside the cell media. The surrounding quartz glass walls are marked gray, the fluid medium white. At the inlet position (*L*/*L*_0_ = 0.00), the media temperature is 37 °C. From there it rises while passing the microwave field and reaches an average temperature of 44 °C at the outlet (*L*/*L*_0_ = 1.00). However, not only the fluid temperature is rising under influence of the microwave field but also the quartz glass temperature. As the quartz glass tube is permanently exposed to thermal and microwave load it stores heat despite its low dielectric loss. In the middle of the pipe, at *L*/*L*_0_ = 0.50, the simulations reveal a difference between both microwave frequencies. At 2.45 GHz the quartz glass tube reaches higher wall temperatures than 915 MHz: 45.6 °C compared to 44.3 °C. At the outlet, the wall temperatures are 46.4 °C for 2.45 GHz and 44.6 °C for 915 MHz. Further, there are differences in the distribution of the electric field as shown in Figure 7c,d. The wavy line for the electric field inside the cell media in Figure 7c results from high damping and energy absorption of the media at 2.45 GHz. In contrast, the simulation for 915 MHz shows a fairly constant radial electromagnetic energy distribution inside the cell media indicating a homogeneous energy absorption throughout the tube (Figure 7d). In both systems, the interphase between quartz glass and cell media causes a jump in E-field intensity.

### 3.3. Cell Death Induction by Radiative-Based Hyperthmia and Combinatory Treatment with Radiotherapy in B16-Melanoma and MCF-7 and MDA-MB-231 Breast Cancer Cells

#### 3.3.1. Radiative-Based Hyperthermia at Both 915 MHz and 2.45 GHz Significantly Inactivates Tumor Cells at 44 °C

**Figure 8:**
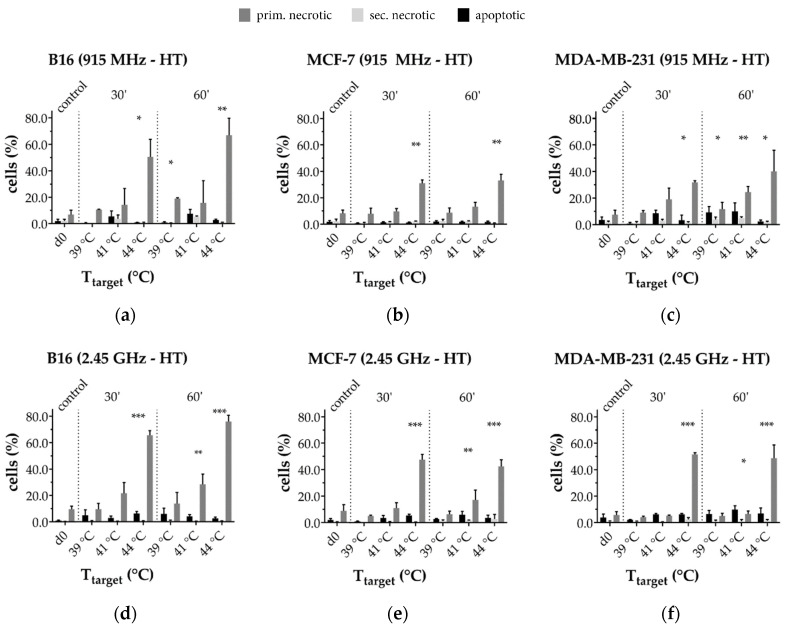
Cell death forms of hyperthermia (HT)-treated cells at 915 MHz (top row) or at 2.45 GHz (bottom row); (**a**,**d**) murine B16 melanoma; (**b**,**e**) human MCF-7 and (**c**,**f**) MDA-MB-231 breast cancer cells. The total percentage of dead cells yields the tumor cell killing efficiency. Mean ± S.D. are presented from at least three independent experiments, each measured in duplicates. Significance was conducted using Kruskal-Wallis test with Dunn’s correction multiple comparison, by comparing treatment related total percentage of inactivated cells to the corresponding control of mock-treated cells at the indicated time point (d0); * (*p* < 0.1), ** (*p* < 0.01), *** (*p* < 0.001).

As shown in Figure 8, circulating the tumor cells at a physiological temperature of 37 °C (control) in the 915 MHz (top row) or 2.45 GHz (bottom row) closed-loop system setup does not influence cell viability, which is ≥90%. The highest rates of dead cells, i.e., the sum of apoptotic (AxV^+^/PI^-^), primary (AxV^+^/PI^++^) and secondary necrotic (AxV^+^/PI^+^) cells, were found at *T*_target_ = 44 °C. By comparing the effective treatment times of 30 min (30’) with 60 min (60’), no significant difference was found for all examined cell lines and temperatures.

Furthermore, a cell line- and frequency-dependent inactivation behavior was found. B16 melanoma showed the highest degree of inactivation at 44 °C with about 80% at 2.45 GHz (Figure 8d) and 70% at 915 MHz (Figure 8a). The percentage of dead cells at 2.45 GHz and 44 °C in the human breast cancer cell lines was 50% for MCF-7, and 60% for MDA-MB-231, while it was approximately 15% lower at 915 MHz.

#### 3.3.2. In a Long-Term Follow-Up, at Temperatures of 39 °C and 41 °C Induced by 915 MHz Hyperthermia, Radiotherapy Is the Main Cell Death Inducer, While at 44 °C, It Is the Heat Application

**Figure 9:**
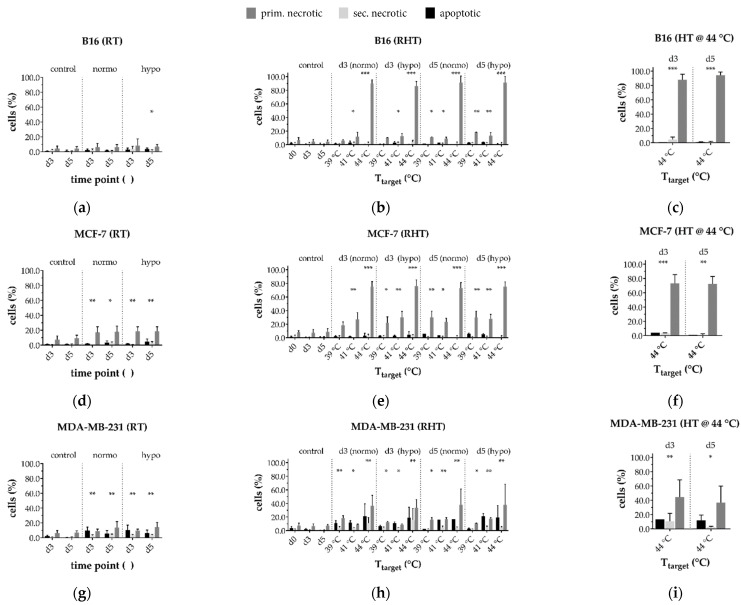
Cell death forms of (**a**,**b**,**c**) B16 melanoma; (**d**,**e**,**f**) MCF-7 breast cancer and (**g**,**h**,**i**) MDA-MB-231 breast cancer after radiotherapy (RT, left column) alone or in combination with normo- or hypofractionation radiotherapy (RHT) on day 3 (d3) and day 5 (d5) (middle column). The right column shows cell death forms for 915 MHz hyperthermia at 44 °C on d3 and d5. The total percentage of dead cells yields the tumor cell killing efficiency. Mean ± S.D. are presented from at least three independent experiments, each measured in duplicate. Significance was conducted using Kruskal-Wallis test with Dunn’s correction multiple comparison, by comparing treatment related total percentage of inactivated cells to the corresponding control of mock-treated cells at the indicated time point (d3, d5); * (*p* < 0.1), ** (*p* < 0.01), *** (*p* < 0.001).

As hyperthermia should be combined with established treatment methods such as radiotherapy, we investigated the combined implementation. To find out how the individual treatment methods affect cell inactivation in the follow-up, i.e., on day 3 (*d*3) and 5 (*d*5), we first analyzed forms of cell death after radiotherapy only (Figure 9a,d,g). B16 cells (Figure 9a) were hardly affected by RT. MCF-7 (Figure 9d) and MDA-MB-231 (Figure 9g) in contrast, showed higher sensitivity to ionizing radiation, resulting in significant cell death rates upon both normo- and hypofractionation on *d*3 and *d*5. The caspase-3 deficient MCF-7 cells showed less apoptosis.

For the combined treatment with 915 MHz (Figure 9b,e,h), the highest levels of dead cells were found at 44 °C, both on *d*3 and *d*5. At 44 °C cell death is dominated by the heating effect (Figure 9c,f,i), especially for B16 (Figure 9b) and MCF-7 (Figure 9e), while MDA-MB-231 (Figure 9h) showed 10–15% lower inactivation rates compared to MCF-7. In contrast to standalone hyperthermia, significant inactivation was achieved for all three cell lines at lower target temperatures such as 39 °C and 41 °C. Independent of the type of treatment, MDA-MB-231 showed a significant portion of apoptotic cells on *d*3 and *d*5 unlike the caspase-3-deficient MCF-7.

### 3.4. Hyperthermia with 915 MHz Barely Impacts on the Immune Checkpoint Molecule Expression

**Figure 10:**
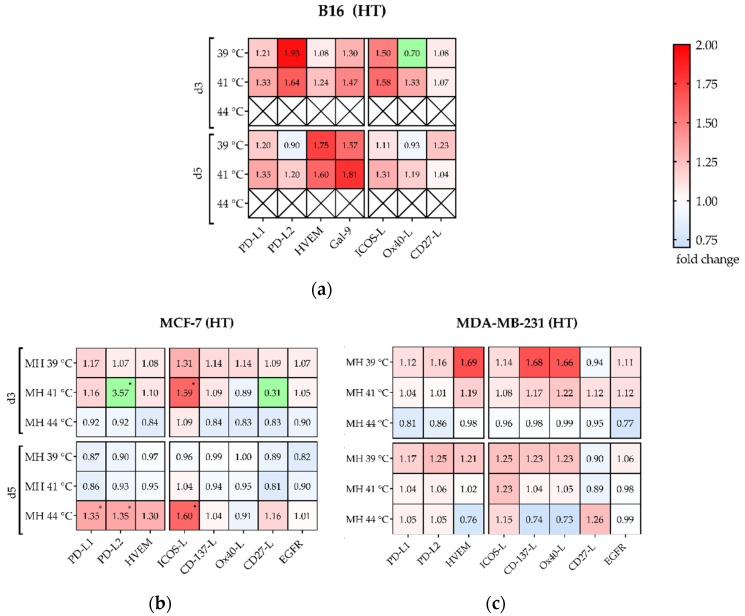
Heatmap of normalized expression (change in mean fluorescence intensity compared to mock-treated cells) of immune checkpoint molecules and of EGFR on day 3 (*d*3) and day 5 (*d*5) on the cell surface of (**a**) B16 melanoma; (**b**) MCF-7 breast cancer and (**c**) MDA-MB-231 breast cancer cells after 915 MHz hyperthermia at 39 °C, 41 °C or 44 °C. Green boxes are values of ∆MFI < 0.75 or > 2.0. Significance test was conducted using Kruskal-Wallis test with corrected Dunn’s multiple comparison from three independent experiments, each measured in duplicates. * (*p* < 0.1).

As shown by various in vitro, in vivo and phase I-III trials, hyperthermia can modulate the immune response, e.g., by influencing the release of the danger signal heat shock protein 70 (HSP70) [27,28]. So far, a lack of data is noted on expression of either immunosuppressive, or immunostimulatory ICM on cancer cells after treatment with HT at 915 MHz. Therefore, murine B16 melanoma (Figure 10a), human MCF-7 (Figure 10b) and MDA-MB-231 (Figure 10c) breast cancer cells were investigated after exposition to 39 °C, 41 °C and 44 °C for 60 min using 915 MHz. Standalone hyperthermia barely showed modulation of prominent ICM. Only PD-L2 and ICOS-L showed a significant increase on MCF-7 cells (Figure 10b) at 41 °C on *d*3, as well as at 44 °C on *d*5. Interestingly, various immune checkpoint molecules were downregulated, especially for MCF-7 on *d*3 at 44 °C, and on *d*5 at 39 °C and 41 °C. For B16 (Figure 10a), no significant modulation of ICMs was found at 39 °C and 41 °C. Since only viable cells were selected for analysis but almost all cells were killed at 44 °C, the data series for 44 °C is excluded here. In summary, standalone HT at 915 MHz does not have strong influence on ICM expression in all examined cell lines.

#### Significant Increase of Several Immune Checkpoint Molecules by Multimodal Treatment Using 915 MHz Hyperthermia in Combination with Radiotherapy

**Figure 11:**
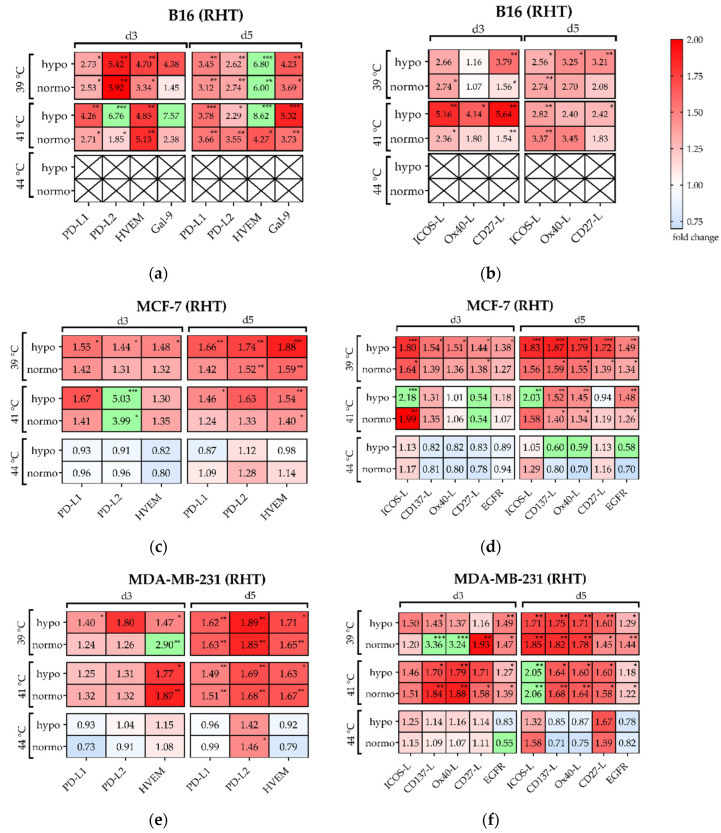
Heatmap of normalized expression (change in mean fluorescence intensity compared to mock-treated cells) of immune checkpoint molecules and of EGFR on day 3 (*d*3) and day 5 (*d*5) on the cell surface of (**a**,**b**) B16 melanoma and (**c**,**d**) MCF-7 breast cancer and (**e**,**f**) MDA-MB-231 breast cancer cells after hyperthermia at 39 °C, 41 °C or 44 °C and normo- and hypo-fractionated radiotherapy. Suppressive immune checkpoint molecules (PD-L1, PD-L2, HVEM) are presented in (**a**,**c**,**e**), while stimulatory immune checkpoint molecules (ICOS-L, CD137-L, Ox40-L, CD27-L) and EGFR are presented in (**b**,**d**,**f**). Green boxes are values of ∆MFI <0.70 or ≥6.0 (a,b), and ∆MFI <0.70 or >2.0 (c-f). Significance test was conducted using Kruskal-Wallis test with corrected Dunn’s multiple comparison from three independent experiments, each measured in duplicate. * (*p* < 0.1), ** (*p* < 0.01), *** (*p* < 0.001).

The Antonia trial on NSCLC gave the first hints of the dynamic modulation of the ICM PD-1/PD-L1-axis, and also identified a significant positive effect when radiotherapy was applied to the immunotherapeutic agent (Durvalumab^®®^) within a short time frame (<14 days vs. >14 days) [29]. Therefore, we analyzed the expression of prominent ICM on day 3 and 5 after combined treatment with hyperthermia at 915 MHz and radiotherapy. A dynamic modulation of immune suppressive ICM such as PD-L1, PD-L2, HVEM and Galectin-9 (only on B16 melanoma) and immune stimulatory ICM like ICOS-L, CD137-L, Ox40-L, and CD27-L on both murine and human cancer cells was observed (Figure 11). While the expression of the immunosuppressive PD-L1 (Figure 11a,c,e) showed a moderate increase in all cell lines after treatment at 39 °C or 41 °C on *d*3 and *d*5, the expression of PD-L2, HVEM and Gal-9 was even more upregulated. This was especially found for PD-L2 and Gal9 in B16 (Figure 11a) on both sampling days and mostly independent of target temperature and irradiation scheme. A similar effect was found for MCF-7 at 41 °C on *d*3 (Figure 11c). In contrast, the expression of immunosuppressive immune checkpoint molecules was almost unchanged for both human breast cancer cell lines at 44 °C (B16 at 44 °C is again excluded due almost complete cell inactivation).

Regarding immune stimulatory ICM (Figure 11b,d,f), treatment and cell line specific modulations were found. For both 39 °C and 41 °C, different ICMs were upregulated by the combinatorial treatment (RHT), while it was sometimes more on day 5, e.g., for MDA-MB-231 at 39 °C and hypofractionation radiation (Figure 11f). Similar to the immunosuppressive ICM, the immunostimulatory ICM were almost unchanged at 44 °C in combination with radiotherapy, and in some cases even downregulated.

### 3.5. Local Absorption in Cell Membrane Components Show Frequency Dependent Correlations

The simulation results described above are a first approach to explain the biological differences found in our biological experiments, with slightly higher inactivation rates for the frequency of 2.45 GHz. However, it does not allow a deep insight into interactions of electromagnetic waves with single cells on the submicron scale. Therefore, we investigate the rate of absorption at the microscopic level, i.e., cell level, at various frequencies and cell radii (4 to 14 µm). The foundations of this approach are described elsewhere [25].

**Figure 12:**
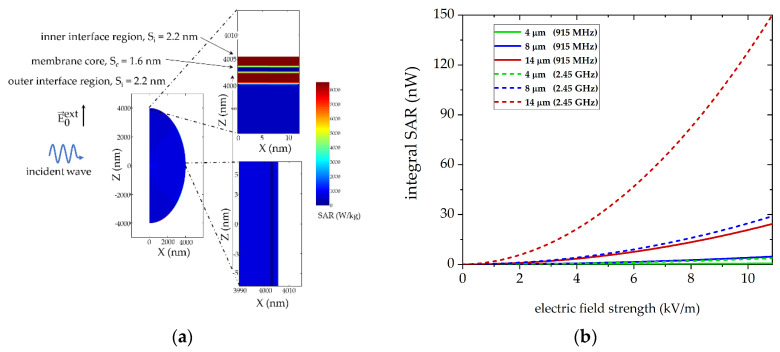

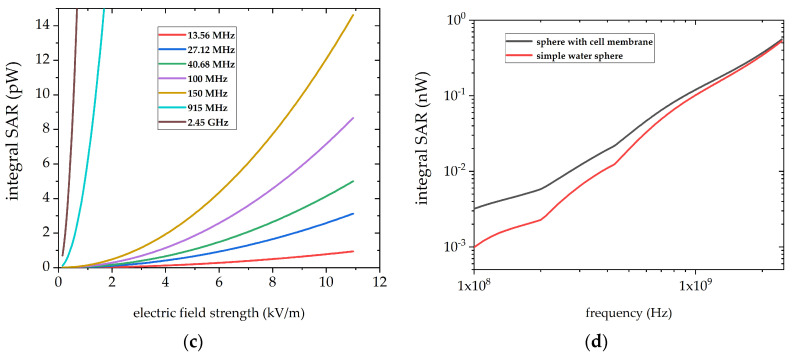
Specific absorption rate (SAR) of a (**a**) small spherical cell with an outer radius of *R* = 4 μm and a phospholipid bilayer with outer interface region (*S*_i_ = 2.2 nm), membrane core (*S*_c_ = 1.6 nm) and inner interface region (*S*_i_ = 2.2 nm) at hyperthermia relevant frequency of 2.45 GHz and an electric field strength of 1000 V/m; (**b**) Integral SAR at frequencies of 915 MHz (solid line) and 2.45 GHz (dashed lines) at three different cell radii (4, 8 and 14 μm) and variable electric field strength; (**c**) Integral SAR for a 4 μm cell depending on the electric field strength and ISM relevant frequencies; (**d**) Comparison of the integral specific absorption rate for a water sphere and a model cell with radius 4 μm in an electric field with 4 kV/m field strength and variable frequency.

As real cellular components such as cell membranes or whole cells are very complex structures, we use a spherical model of a cell with radius 4 μm to 14 μm. The cell has a simplified structure consisting of a cytoplasm region (*R*_cyto_ = 10 nm) that is enclosed by a single DPPD-bilayer structure (*S*_bilayer_ = 6 nm) and a solvent region that is 10^5^ times larger than *R*_cyto_. Figure 12a illustrates the microwave energy absorption at 2.45 GHz of a simplified cell exposed to an electric field with 1000 V/m. Most of the electromagnetic energy is absorbed in the DPPD-bilayer, especially of the inner interface region (SAR values are up to eight times higher than for the outer interface). Interestingly, a strong angle dependence with maxima in the equatorial region was found. As described in Section 2.5 we aimed to deduce the local SAR values of the cell at a global SAR value in order to link microscopic and macroscopic calculations. Figure 12b shows the global SAR values over electric field strength at 915 MHz and 2.45 GHz for different cell radii. This clearly shows a size-depending effect in global SAR values. The smaller the cell, the less energy is absorbed, especially at 2.45 GHz. Further, power adsorption is much higher at 2.45 GHz compared to 915 MHz. This tendency is confirmed in Figure 12c**,** illustrating global SAR values for a cell with 4 μm at various microwave and radio frequencies. Global SAR values drop with decreasing frequencies. As mentioned above, our model is very simple as it lacks certain cell components. However, the model demonstrates the role of the DPPD-bilayer and frequency for a single cell’s energy absorption. Figure 12d clarifies the influence, comparing the model cell’s energy absorption with a sphere consisting of just pure water. The existence of a membrane structure (DPPD bilayer) significantly increased power adsorption, especially below 10^9^ Hz. Further, Figure 12d shows that the SAR value of a single cell drops from 0.6 nW at 2.45 GHz to 0.1 nW at 915 MHz.

## 4. Discussion

### 4.1. In Silico Simulations on Cells and Biomacromolecules Are Helpful to Describe Effects of Non-Ionizing Electromagnetic Radiation

Interactions between biological systems such as biomacromolecules or cells and electromagnetic radiation have been studied for decades. In particular, results of non-ionizing radiation below the UV light range (<750 THz or >400 nm) are sometimes controversial, often due to the inaccurate description of the experimental setup including the temperature distribution and possible hot-spots in the macro- and submicron range. AC current application (<300 Hz) has proven to stimulate non-thermal effects and affect DNA synthesis, as demonstrated by Tsai et al. in the proliferation behavior of osteoblasts [30,31,32]. At radio and microwave frequencies (RF) at 3 MHz to 300 GHz the heat development in the cell rises due to alternating polarization, rotation and torsion of molecules, until the thermal effect dominates the stimulating effect [30]. The rise in temperature ultimately leads to an impairment of cellular functions and cell damage [33]. The energy applied by electromagnetic radiation is comparable to the amount of energy required for protein denaturation [34]. Still, the question is whether RF might cause significant modifications in a biological system without measurable temperature increase [30,35]. Temperature-independent effects could be assigned to resonant vibrations of large molecules and other biocomponents, e.g., in microtubules [30,33,36]. In addition, there may be an interaction of RF with the magnetic spin of radical pairs; this slows down the decay of the radical pair’s spin, which extends its lifespan. For example, high concentrations of reactive oxygen species (ROS) cause damage in the cell membrane, in the DNA as well as in the conformation and linkage of proteins [30].

Our simulations for a simplified single cell model show that the SAR of the outer monolayer of the DPPD is significantly higher, while the inner monolayer is shielded by the bilayer’s core. This could be a first hint as to why radiofrequency inactivates cells at a much higher magnitude at 44 °C in comparison to conventional warm-water, as described by our group in previous experiments [14]. To obtain more information about the interactions between electromagnetic radiation and cells, future simulation models should therefore consider transmembrane proteins and other essential building blocks such as DNA, mitochondria or enzymes. First results on nucleosides already show a similar frequency dependence as for DPPD [37]. Furthermore, our single-cell simulations show that the integral absorption at 2.45 GHz is more than 550% higher than at 915 MHz. This is in contrast to the macroscopic simulations of the two heating units. These show that the penetration depth increases with lower frequency. At the same time, however, the electric field strength remains at a similarly high level. This indicates that (non)thermal effects originate in the sub-micrometer range. Therefore, in future experiments with our modular circuit system, instead of heating cells, for example, proteins, enzymes or lipid spheres should be heated to provide a detailed insight into cell component interactions with RF.

### 4.2. A Mixed Temperature Profile during Hyperthermia in Combination with Radiotherapy Could Foster Anti-Tumor Immunreactions and Effectiveness of Immune Checkpoint Molecule Drugs

Hyperthermia is probably the oldest method in the fight against cancer. Its immunomodulatory properties have been investigated and demonstrated in various in vitro, in vivo and phase I-III studies [4,38]. For example, Schildkopf et al. demonstrated in in vitro experiments on the human colorectal tumor cell lines HCT15 and SW480 and the mouse colon carcinoma cell line CT26-WT that temperatures above 41 °C in combination with radiotherapy resulted in significantly increased proinflammatory cytokine secretion (IL-8 and IL-12), and increased phagocytosis rates of macrophages and DCs compared with radiotherapy alone [27]. These cytotoxic effects of additional hyperthermia are related, in part, to the denaturation of various proteins [39]. Protein denaturation also stimulates the heat-induced synthesis of so-called heat shock proteins (HSPs). HSPs are relevant for protein folding and transport as well as for the formation of protein quaternary structures. Therefore, HSPs counteract protein denaturation and induce some heat tolerance. Outside the cell, however, they are immune activating. In vivo, the thermal sensitivity of cells is not constant but strongly depends on the microenvironment. Influencing factors are the pH and the supply of oxygen and nutrients to the tissue, which is lower in malignant tumors due to their chaotic vascularization. In addition, the poor blood supply to the tumor tissue leads to lower heat dissipation and thus to higher temperatures. Finally, hyperthermia can lead to increased antigen presentation including recruitment and activation of T lymphocytes [40,41,42]. Clinical trials, such as the phase III trial in high-risk soft tissue sarcoma by Issels et al. [43], have evaluated neo-adjuvant chemotherapy alone or in combination with regional hyperthermia. Regional hyperthermia was administered on day 1 and 4 of each cycle for 60 min with a target tumor temperature of 42 °C during both induction and post-induction therapy. Regional hyperthermia and thermal mapping were performed according to ESHO quality guidelines and safety assurance [44]. Various parameters were used to quantify the treatment temperatures in hyperthermia, such as the maximum temperature (*T*_max_) or the median time-average temperatures reached in 20%, 50%, and 90% of all measured sites (*T*_20_, *T*_50_, and *T*_90_, respectively). In this exemplary phase III study, *T*_max_ was 41.8 °C (41.1–43.2 °C), *T*_20_ was 40.8 °C (40.1–42.3 °C), *T*_50_ was 40.3 °C (39.5–41.0 °C), and *T*_90_ was 39.2 °C (38.5–39.8 °C) [43]. However, the immunobiological effects that occur at these different temperatures and after hyperthermia have not been studied and are mostly absent in clinical hyperthermia studies. Thus, longitudinal blood tests, as described in [45], could help to improve clinical acceptability and provide a more valid immunobiological rationale.

Similar local and systemic effects are known for radiotherapy as for hyperthermia, e.g., HSP70 and ATP release or NK and DC activation [46]. Recently, many clinical trials focus on the combination of RT with immunotherapies, e.g., immune checkpoint molecules, but hyperthermia is rarely included [47]. Immune checkpoint molecules are associated with many mechanisms of immune surveillance, immune editing, and immune escape [48]. For example, the PD-L1/PD-1 axis is an essential regulator of T cell activation, as tumor cells suppress T cell-mediated anti-tumor immune responses through this axis [49]. Targeting immunosuppressive ICM is a promising strategy to improve the anti-tumor immune response of the organism, either as a single treatment or mostly in combination with standard therapies such as RT in a subset of cancer patients [50].

We have developed an in vitro system to measure clinically relevant hyperthermia effects at two frequencies, 915 MHz and 2.45 GHz, and at different target temperatures, 39 °C, 41 °C and 44 °C, alone and in combination with normo- and hypofractionation irradiation, to find out how RT in combination with HT alters the immunogenic phenotype of tumor cells and which combination is most beneficial, depending on the microwaves’ frequency. This may also provide the first clues on how to increase the response rate of additional immunotherapies such as immune checkpoint inhibitors (ICI), e.g., by optimizing treatment protocols and taking further into account the individual patient’s tumor [3,50,51,52].

In addition to the frequency dependence, a cell line difference was found, i.e., murine B16 melanoma had a higher inactivation rate by microwave-based HT compared with human MCF-7 and MDA-MB 231 breast cancer cells. A possible explanation could be differences in lipid membrane composition, which causes lower stability under thermal stress [51]. In general, tumor cells are more heat sensitive than normal cells, which is beneficial for the survival of healthy tissues and immune cells during hyperthermia [52]. Further experiments with tumor cells in combination with cells of the peripheral blood could be performed within our closed-loop system.

**Figure 13:**
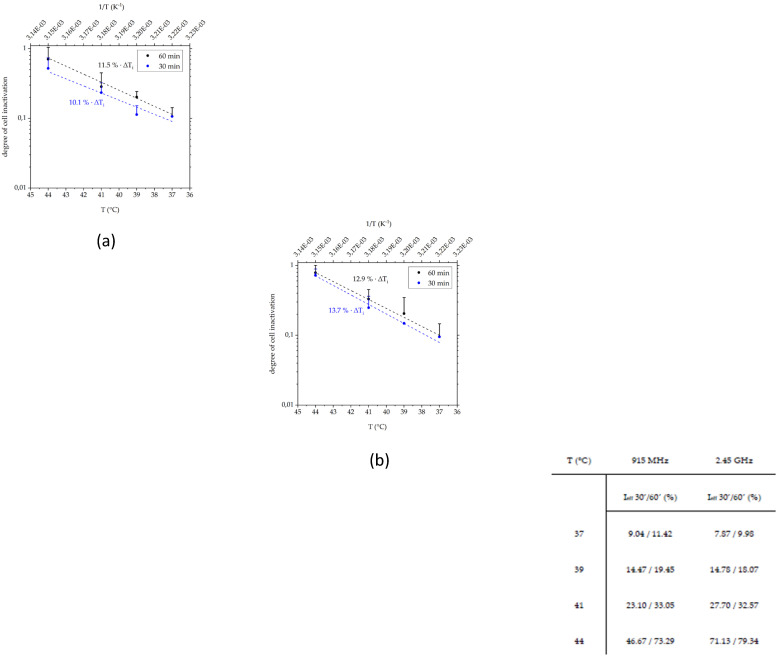
Arrhenius plot for heat inactivation efficiency (I_eff_) of B16 melanoma cells at (**a**) 915 MHz and (**b**) 2.45 GHz at time points 30 min (30′, blue dashed line) and 60 min (60′, black dashed line) in the temperature range between 37 °C and 44 °C. Accompanying inactivation efficiency was calculated by linear regression showing *I*_eff_ at different temperatures.

In particular at 44 °C, heating was the dominant cell death inductor at both frequencies. Interestingly, 30 min of heat showed nearly similar inactivation efficiency as it was found for 60 min. This is especially important during hyperthermia treatment when an early stop is required, e.g., because of pain or discomfort [53]. To optimize and provide a tool for predicting inactivation efficiency and temperature-related biological responses, e.g., for HSP70 promoter activation [54], our closed-loop system is useful. Using our inactivation efficiency data in a semi-logarithmic modified version of Arrhenius inactivation law [52], we were able to predict the cell-line specific inactivation efficiency in a time-, temperature- and frequency-dependent manner (Figure 13a,b). For hyperthermia at 915 MHz (Figure 13a), the percentage of inactivated B16 cells increased by 11.5% per 1 °C (∆*T*_i_) for 60 min and 10.1% for 30 min, respectively, while at 2.45 GHz (Figure 13b) it was 12.9% for 60 min and 13.7% for 30 min. It should be noted that this linearity was found only in the experimental temperature range between 37 °C and 44 °C. During hyperthermia, the cumulative equivalent minute dose (CEM43 °C) is often set as a non-linear breakpoint [55], which was also found in the inactivation efficiency for mammalian cell lines by Dewey et al. [52]. Using our system in combination with experimental data, an estimation of the inactivation efficiency and other immunobiological processes could be made by an additional comparison with temperature-based MRI images from clinical treatment [10,53].

### 4.3. Preclinical Experiments Open New Therapeutic Fields and Show a Highly Dynamic Expression of Immune Checkpoint Molecules

Prognostic and predictive markers are the basis for the decision for or against radio(chemo)therapy, endocrine therapy, and/or targeted therapies, e.g., hormones or immune checkpoint inhibitors (ICI). The latter have changed the field of oncology in many ways, especially in advanced or metastatic tumors such as non-small cell lung cancer (NSCLC), melanoma, or triple-negative breast cancer (TNBC) [56]. A standard for deciding which combination of ICI should be used, tumor and tumor-associated immune cell staging is mandatory but staining and counting methods including cut-off values are still not standardized and more or less empirically chosen. Another important issue is the heterogeneity of the biomaterial itself. For example, the biopsies were taken from the primary tumor or metastatic tumor and previously collected as part of a treatment option [57]. There may be differences in immune cell population composition and PD-L1 status, especially for the tumor itself [4,58].

It is generally accepted that RT affects the expression of PD-L1 [49,59,60], but a detailed understanding of the expression of other ICM within the immunological synapse after exposure to RT, HT, or a combination of both methods is not available [4]. For the B16 melanoma cell line and the two human breast cancer cell lines, MDA-MB-231 and MCF-7, we found that hyperthermia alone had no significant effect on the expression of both immunosuppressive (PD-L1, PD-L2, HVEM and Gal-9) and immunostimulatory (ICOS-L, CD137-L, Ox40-L, CD27-L) checkpoint molecules and of EGFR. In previous experiments, for the two human breast cancer cell lines, we found that RT not only increased the expression of PD-L1 on the tumor cell surface, but also of PD-L2 and HVEM and other immune checkpoint stimulatory inhibitors in a time- and cell line-dependent manner [14]. For the murine B16 melanoma cell line, this dynamic modulation by RT alone can be confirmed, while HVEM showed the highest fold-change in upregulation. Interestingly, for human melanoma (*n* = 116), Malissen et al. also found HVEM to have broader expression than PD-L1 and thus suggest more focus on HVEM as a prognostic marker and potential treatment target [61]. The addition of HT to RT, which may open a new therapeutic option to improve the response rate to immune checkpoint inhibitor drugs, showed the highest upregulation in all three cell lines. This was not directly related to a specific hyperthermia temperature.

In summary, biological variants in tumor tissue are normal [62,63,64] and should be monitored in detail for prognosis, prediction, and treatment adaptation in multimodality therapies [65,66]. Thus, patient-specific selection of ICI allows for better clinical outcome and improves the acceptability of adding hyperthermia to radiotherapy. However, not only immunosuppressive checkpoint molecules showed increased expression after RHT, but also immunostimulatory molelcules such as CD137-L, Ox40-L, CD27-L, and ICOS-L were individually affected depending on the tumor cells, temperature, and mode of application of HT. This requires early and repeated analysis of each patient’s tumor tissue or associated accessible biomaterial for treatment planning and adjustment [4].

## 5. Conclusions

A new focus in preclinical and clinical studies needs to be placed on the combination of hyperthermia and radiotherapy, i.e., temperature, frequency, tumor entity, and timing within multimodal tumor treatments. Our in-silico simulations showed frequency-dependent energy absorption by cellular components, which is currently not considered in hyperthermia. In addition, differences between heating approaches, e.g., by electromagnetic radiation, infrared, or conventional warm-water, could be better understood. The expression of immune checkpoint molecules was highly dynamic and needs to be studied in much more detail to optimize targeted and personalized drugs in the future. The ex vivo results obtained with our system and adapted devices may provide initial clues for tailoring patient-specific treatment. Key analyses of the immunogenic properties of the tumor cells following the treatments by determining apoptosis, primary and secondary necrosis, the latter two tumor cell death forms being indicators for high immunogenicity, were performed in this work. Additionally, the tumor cell surface composition regarding the expression of immune checkpoint molecules was characterized. Future work should additionally include functional immune cells assays, such as testing the activation and T cell stimulating properties of dendritic cells after being in contact with the treated tumor cells [67]. Further, vaccination and therapeutic preclinical in vivo experiments should be performed to obtain final conclusions about the best immunogenic treatment, consisting of microwave-based HT with RT [68]. In this regard, in vivo studies with both immunocompetent and immunodeficient mice in multimodal treatments, i.e., radio(chemo)therapy and immunotherapy, in combination with histological analyses and time-dependent immune monitoring should be performed.

## Figures and Tables

**Table 1:** Complex relative permittivity ε_r_ = $\varepsilon_{r}^{\prime }$ − j$\varepsilon_{r}^{\prime \prime }$ of pure water at 915 MHz [15] or 2.45 GHz [16] as a function of temperature.

Parameter	25 °C	35 °C	45 °C	55 °C
$\varepsilon_{r}^{\prime }\left(T\right)$	81.26 ^1^/76.70 ^2^	75.77 ^1^/74.00 ^2^	71.67 ^1^/70.70 ^2^	68.40 ^1^/67.50 ^2^
$\varepsilon_{r}^{\prime \prime }\left(T\right)$	3.82 ^1^/12.04 ^2^	3.39 ^1^/9.40 ^2^	3.09 ^1^/7.50 ^2^	2.89 ^1^/6.01 ^2^

^1^ for 915 MHz, ^2^ for 2.45 GHz

**Table 2:** List of antibodies used for the analysis of the surface expression of ICM and of EGFR on human and murine cancer cells by multicolor flow cytometry.

Marker	Mastermix #1, Human (µL/Well)	Mastermix #2, Human (µL/Well)	Mastermix #1, Murine (µL/Well)	Mastermix #2, Murine (µL/Well)
PD-L1 (CD274)	0.5		0.05	
PD-L2 (CD273)	0.5		0.1	
ICOS-L (CD275)	0.5		0.2	
EGF-Receptor	0.5		not detectable	
HVEM (CD270)		0.5		0.05
Ox40-L (CD134)		0.5	0.05	
TNFRSF9 (CD137-L)		0.5		not detectable
CD70 (CD27-L)		0.5		0.2
Galectin 9				0.05
Zombie NIR	0.1	0.1	0.1	
Zombie Yellow				0.1
FACS buffer (2% FCS in DPBS)	97.9	97.9	99.5	99.6

## Data Availability

The data presented in this study are available on reasonable request from the corresponding author.
